# Interleukin-6 and statin therapy: potential role in the management of COPD

**DOI:** 10.1186/1465-9921-14-74

**Published:** 2013-07-17

**Authors:** Robert P Young, Raewyn J Hopkins

**Affiliations:** 1 School of Biological Sciences and Faculty of Medical and Health Sciences, University of Auckland, Auckland, New Zealand

## Dear Editor

We read with interest the article by Ferrari and colleagues showing in a small prospective study of chronic obstructive pulmonary disease (COPD) patients that interleukin-6 (IL-6) is a useful biomarker predicting worsening exercise tolerance and greater mortality [1]. We outline below the significance of this finding and its potential impact on the future management of COPD.

**Figure 1:**
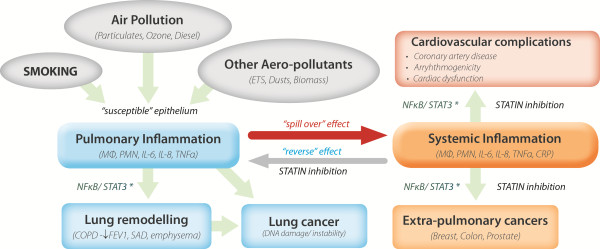
Proposed relationship between Interleukin-6 mediated systemic inflammation, pulmonary inflammation, COPD and COPD co-morbidities.

The findings by Ferrari and colleagues concur with other prospective studies, including the recently published ECLIPSE study, showing elevated IL-6 is a clinically useful marker predicting poor outcomes in COPD [2-4]. In large prospective studies a similar utility was found for elevated C-reactive protein (CRP), whose expression is controlled primarily by IL-6 and also associated with poor outcomes in COPD [5-7]. These poor outcomes include poorer exercise tolerance, worsening lung function, greater exacerbation rate, greater co-morbidities (lung cancer, pneumonia, diabetes and coronary artery disease) and most importantly, greater mortality. Collectively, these findings suggest that the natural history of COPD and its prognosis can be predicted to some extent by elevated IL-6, a serum marker of systemic inflammation. These observations also suggest that systemic inflammation plays a primary pathogenic role in the natural history of this disease (“reverse” effect, Figure 1), not just a secondary phenomenon from pulmonary inflammation (the “spill over” effect, Figure 1) [8]. In animal models where IL-6 is over-expressed, the clinical phenotype closely resembles that of COPD further suggesting a primary role for IL-6 (and systemic inflammation) in the development of COPD [9], We have recently reviewed the literature and suggest that IL-6-mediated systemic inflammation is also relevant to many of the COPD-related co-morbidities described above [8]. The prospective study by Ferrari and colleagues provides further data to suggest that elevated IL-6 plays an active role in the progression of this important disease [1].

If these observations are true, then it follows that HMGCoA reductase inhibitors (statins) might be benefical in COPD patients through their powerful inhibition of IL-6-mediated systemic inflammation [8]. Indeed, there is a large body of data from numerous observational studies showing that statin therapy reduces both morbidity and mortality in COPD including; reducing the rate of infective exacerbations, slowing the decline in FEV_1_, reducing mortality from pneumonia or infective exacerbations and improving exercise tolerance [8]. This last clinical feature of COPD is very important as it significantly affects quality of life. In the first randomized control trial of statin therapy in COPD, exercise tolerance was improved by nearly 50% after 6 months of statin therapy compared to placebo [10]. This improvement correlated with a significant reduction in serum IL-6 level (and CRP) but not lung function [10], suggesting IL-6-mediated systemic inflammation might be one of the primary determinants of poor exercise tolerance. Also of considerable importance, is the recent finding that elevated IL-6 or CRP levels are associated with increased risk of lung cancer [11], particularly in patients with COPD [12], and that lung cancer mortality is reduced by 17% with statin use [Supplementary Figure S11 from ref. [13]. Together with the findings of the observational studies described above, these results make a strong argument for examining the role of statins as adjunct therapy to inhaler therapy in COPD (Figure 1) [8,14]. This is particularly the case as current inhaler therapy in COPD is symptom-based, minimizing breathlessness and reducing exacerbations, while statin-based systemic therapy, inhibiting both systemic and pulmonary inflammation, appears to confer significant disease modifying benefits. It also argues in favor of investigating the utility of measuring serum IL-6 (or it’s surrogate CRP) in patients with COPD to target and monitor therapy [1-7,14].

We conclude that the study of Ferrari and colleagues confirms earlier studies showing that outcomes in COPD are related to IL-6-mediated systemic inflammation [1]. This observation not only provides the basis on which to better phenotype patients with COPD [14], but more importantly highlights the important potential utility of statin therapy as a significant disease-modifying therapy in COPD [8]. This hypothesis requires urgent examination in clinical trials.

## References

[B1] Ferrari R Tanni SE Caram LMO Three-year follow-up of interleukin 6 and C-reactive protein in chronic obstructive pulmonary disease Thorax 2013 68 691 694 10.1186/1465-9921-14-24 23425215 PMC3620569

[B2] Pinto-Plata V Casanova C Mullerova H Inflammatory and repair serum biomarker pattern. Association to clinical outcomes in COPD Respir Res 2012 13 71 10.1186/1465-9921-13-71 22906131 PMC3493287

[B3] Celli BR Locantore N Yates J Inflammatory biomarkers improve clinical prediction of mortality in chronic obstructive pulmonary disease Am J Respir Crit Care Med 2012 185 1065 1072 10.1164/rccm.201110-1792OC 22427534

[B4] Agusti A Edwards LD Rennard SI Persistent systemic inflammation is associated with poor clinical outcomes in COPD: a novel phenotype Plos One 2012 7 e3y483 10.1371/journal.pone.0037483 PMC3356313 22624038

[B5] Man SFP Connett JE Anthonisen NR C-reactive protein and mortality in mild to moderate chronic obstructive pulmonary disease Thorax 2006 61 849 853 10.1136/thx.2006.059808 16738034 PMC2104755

[B6] Dahl M Vestbo J Lange P C-reactive protein as a predictor of prognosis in chronic obstructive pulmonary disease Am J Respir Crit Care Med 2007 175 250 255 10.1164/rccm.200605-713OC 17053205

[B7] Walter RE Wilk JB Larson MG Systemic inflammation and COPD: the Framingham Heart Study Chest 2008 133 19 25 10.1378/chest.07-0058 17908709

[B8] Young RP Hopkins R Eaton TE Pharmacological actions of statins: potential utility in COPD Eur Respir Rev 2009 118 222 232 20956147 10.1183/09059180.00005309

[B9] Kuhn C Homer RJ Zhu Z Airway responsiveness and airway obstruction in transgenic mice: morphologic correlates in mice over-expressing IL-11 and IL-6 in the lung Am J Respir Cell Mol Biol 2000 22 289 295 10.1165/ajrcmb.22.3.3690 10696065

[B10] Lee T-M Lin M-S Chang N-C Usefulness of C-reactive protein and interleukin-6 as predictors of outcomes in patients with chronic obstructive pulmonary disease receiving pravastatin Am J Cardiol 2008 101 530 535 10.1016/j.amjcard.2007.09.102 18312772

[B11] Young-Yin K Young-Min K Kyae HK High-sensitivity C-reactive protein levels and cancer mortality Cancer Epidemiol Bio Prev 2012 21 2076 2086 10.1158/1055-9965.EPI-12-0611 23136255

[B12] Thomsen M Dahl M Lange P Inflammatory biomarkers in chronic obstructive pulmonary disease Am J Respir Crit Med 2012 186 982 988 10.1164/rccm.201206-1113OC 22983959

[B13] Nielson SF Nordestggard BG Bojesin SE Statin use and reduced cancer-related mortality N Eng J Med 2012 367 1792 1802 10.1056/NEJMoa1201735 23134381

[B14] McDonald VM Higgins I Woods LG Gibson PG Multidimensional assessment and tailored interventions for COPD: respiratory utopia or common sense? Thorax 2013 10.1136/thoraxjnl-2012-202646 PMC3711365 23503624

